# Applying unmixing to gene expression data for tumor phylogeny inference

**DOI:** 10.1186/1471-2105-11-42

**Published:** 2010-01-20

**Authors:** Russell Schwartz, Stanley E Shackney

**Affiliations:** 1 Department of Biological Sciences, Carnegie Mellon University, Pittsburgh, PA USA; 2 Departments of Human Oncology and Human Genetics, Drexel University School of Medicine, Pittsburgh, PA USA

## Abstract

**Background:**

While in principle a seemingly infinite variety of combinations of mutations could result in tumor development, in practice it appears that most human cancers fall into a relatively small number of "sub-types," each characterized a roughly equivalent sequence of mutations by which it progresses in different patients. There is currently great interest in identifying the common sub-types and applying them to the development of diagnostics or therapeutics. Phylogenetic methods have shown great promise for inferring common patterns of tumor progression, but suffer from limits of the technologies available for assaying differences between and within tumors. One approach to tumor phylogenetics uses differences between single cells within tumors, gaining valuable information about intra-tumor heterogeneity but allowing only a few markers per cell. An alternative approach uses tissue-wide measures of whole tumors to provide a detailed picture of averaged tumor state but at the cost of losing information about intra-tumor heterogeneity.

**Results:**

The present work applies "unmixing" methods, which separate complex data sets into combinations of simpler components, to attempt to gain advantages of both tissue-wide and single-cell approaches to cancer phylogenetics. We develop an unmixing method to infer recurring cell states from microarray measurements of tumor populations and use the inferred mixtures of states in individual tumors to identify possible evolutionary relationships among tumor cells. Validation on simulated data shows the method can accurately separate small numbers of cell states and infer phylogenetic relationships among them. Application to a lung cancer dataset shows that the method can identify cell states corresponding to common lung tumor types and suggest possible evolutionary relationships among them that show good correspondence with our current understanding of lung tumor development.

**Conclusions:**

Unmixing methods provide a way to make use of both intra-tumor heterogeneity and large probe sets for tumor phylogeny inference, establishing a new avenue towards the construction of detailed, accurate portraits of common tumor sub-types and the mechanisms by which they develop. These reconstructions are likely to have future value in discovering and diagnosing novel cancer sub-types and in identifying targets for therapeutic development.

## Background

One of the great contributions of genomic studies to human health has been to dramatically improve our understanding of the biology of tumor formation and the means by which it can be treated. Our understanding of cancer biology has been radically transformed by new technologies for probing the genome and gene and protein expression profiles of tumors, which have made it possible to identify important sub-types of tumors that may be clinically indistinguishable yet have very different prognoses and responses to treatments [1-4]. A deeper understanding of the particular sequences of genetic abnormalities underlying common tumors has also led to the development of "targeted therapeutics" that treat the specific abnormalities underlying common tumor types [5-7]. Despite the great advances molecular genetics has yielded in cancer treatment, however, we are only beginning to appreciate the full complexity of tumor evolution. There remain large gaps in our knowledge of the molecular basis of cancer and our ability to translate that knowledge into clinical practice. Some recognized sub-types remain poorly defined. For example, multiple studies have identified distinct sets of marker genes for the breast cancer "basal-like" sub-type, which can lead to very different classifications of which tumors belong to the sub-type [2,3,8]. In other cases, there appear to be further subdivisions of the known sub-types that we do not yet understand. For example, the drug traztuzumab was developed specifically to treat the HER2-overexpressing breast cancer sub-type, yet HER2 overexpression as defined by standard clinical guidelines is not found an all patients who respond to traztuzumab, nor do all patients exhibiting HER2 overexpression respond to traztuzumab [9]. Furthermore, many patients do not fall into any currently recognized sub-types. Even when a sub-type and its molecular basis is well characterized, the development of targeted therapeutics like traztuzumab is a difficult and uncertain process with a poor success rate [10]. Clinical treatment of cancer could therefore considerably benefit from new ways of identifying sub-types missed by the prevailing expression clustering approaches, better methods of finding diagnostic signatures of those sub-types, and improved techniques for identifying those genes essential to the pathogenicity of particular sub-types.

More sophisticated computational models of tumor evolution, drawn from the field of phylogenetics, have provided an important tool for identifying and characterizing novel cancer sub-types [11]. The principle behind cancer phylogenetics is simple: tumors are not merely random collections of aberrant cells but rather evolving populations. Computational methods for inferring ancestral relationships in evolving populations should therefore provide valuable insights into cancer progression. Desper et al. [11-13] developed pioneering approaches to inferring tumor phylogenies (or *oncogenetic trees*) using evolutionary distances estimated from the presence or absence of specific mutation events [11], global DNA copy numbers assayed by comparative genomic hybridization (CGH) [12], or microarray gene expression measurements [13]. More involved maximum likelihood models have since been developed to work with similar measurements of tumor state [14]. These approaches all work on the assumption that a global assessment of average tumor status provides a reasonable characterization of one possible state in the progression of a particular cancer sub-type. By treating observed tumors as leaf nodes in a species tree, Desper et al. could apply a variety of methods for phylogenetic tree inference to obtain reasonable models of the major progression pathways by which tumors evolve across a patient population.

An alternative approach to tumor phylogenetics, developed by Pennington et al. [15,16], relies instead on heterogeneity between individual cells within single tumors to identify likely pathways of progression [17-19]. This cell-by-cell approach is based on the assumption that tumors preserve remnants of earlier cell populations as they develop. Any given tumor will therefore consist of a heterogeneous mass of cells at different stages of progression along a common pathway, as well as possibly contamination by healthy cells of various kinds. This conception arose initially from studies using fluourescence in situ hybridization (FISH) to assess copy numbers of DNA probes within individual cells in single tumors. These studies showed that single tumors typically contain multiple populations of cells exhibiting distinct subsets of a common set of mutations, such as successive acquisition of a sequence of mutations or varying degrees of amplification of a single gene [17,18]. These data suggested that as tumors progress, they retain remnant populations of ancestral states along their progression pathways. The most recent evidence from high-throughput resequencing of both primary tumors and metastases from common patients further supports this conclusion, showing that primary tumors contain substantial genetic heterogeneity and indicating that mestastases arise from further differentiation of sub-populations of the primary tumor cells [20]. The earlier FISH studies led to the conclusion that by determining which cell types co-occur within single tumors, one can identify those groups of cell states that likely occur on common progression pathways [19]. Pennington et al. [15,16] developed a probabilistic model of tumor evolution from this intuition to infer likely progression pathways from FISH copy number data. The Pennington et al. model treated tumor evolution as a Steiner tree problem within individual patients, using pooled data from many patients to build a global consensus network describing common evolutionary pathways across a patient population. This cell-by-cell approach to tumor phylogenetics is similar to methods that have been developed for inferring evolution of rapidly evolving pathogens from clonal sequences extracted from multiple patients [21,22].

Each of these two approaches to cancer phylogenetics has advantages, but also significant limitations. The tumor-by-tumor approach has the advantage of allowing assays of many distinct probes per tumor, potentially surveying expression of the complete transcriptome or copy number changes over the complete genome. It does not, however, give one access to the information provided by knowledge of intratumor heterogeneity, such as the existence of transitory cell populations and the patterns by which they co-occur within tumors, that allow for a more detailed and accurate picture of the progression process. The cell-by-cell approach gives one access to this heterogeneity information, but at the cost of allowing only a small number of probes per cell. It thus allows for only relatively crude measures of state using small sets of previously identified markers of progression.

One potential avenue for bridging the gap between these two methodologies is the use of computational methods for mixture type separation, or "unmixing," to infer sample heterogeneity from tissue-wide measurements. In an unmixing problem, one is presented with a set of data points that are each presumed to be a mixture of unknown fractions of several fundamental *components*. Unmixing comes up in numerous contexts in the analysis and visualization of complex datasets and has been independently studied under various names in different communities, including unmixing, "the cocktail problem," "mixture modeling," and "compositional analysis." In the process, it has been addressed by many methods. One common approach relies on classic statistical methods, such as factor analysis [23,24], principal components analysis (PCA) [25], multidimensional scaling (MDS) [26], or more recent elaborations on these methods [27,28]. Mixture models [29], such as the popular Gaussian mixture models, provide an alternative by which one can use more involved machine learning algorithms to fit mixtures of more general families of probability distributions to observed data sets. A third class of method arising from the geosciences, which we favor for the present application, treats unmixing as a geometry problem. This approach views components as vertices of a multi-dimensional solid (a *simplex*) that encloses the observed points [30] making unmixing essentially the problem of inferring the boundaries of the solid from a sample of the points it contains.

The use of similar unmixing methods for tumor samples was pioneered by Billheimer and colleagues [31] for use in enhancing the power of statistical tests on heterogenous tumor samples. The intuition behind this approach is that markers of tumor state, such as expression of key genes, will tend to be diluted because of infiltration from normal cells or different populations of tumor cells. By performing unmixing to identify the underlying cellular components of a tumor, one can more effectively test whether any particular cell state strongly correlates with a particular prognosis or treatment response. A similar technique using hidden Markov models has more recently been applied to copy-number data to correct for contamination of healthy cells in primary tumor samples [32]. These works demonstrate the feasibility of unmixing approaches for separating cell populations in tumor data.

In the present work, we develop a new approach using unmixing of tumor samples to assist in phylogenetic inference of cancer progression pathways. Our unmixing method adapts the geometric approach of Ehrlich and Full [30] to represent unmixing as the problem of placing a polytope of minimum size around a point set representing expression states of tumors. We then use the inferred amounts by which the components are shared by different tumors to perform phylogenetic inference. The method thus follows a similar intuition to that of the prior cell-by-cell phylogenetic methods, assuming that cell states commonly found in the same tumors are likely to lie on common progression pathways. We evaluate the effectiveness of the approach on two sets of simulated data representing different hypothetical mixing scenarios, showing it to be effective at separating several components in the presence of moderate amounts of noise and inferring phylogenetic relationships among them. We then demonstrate the method by application to a set of lung tumor microarray samples [33]. Results on these data show the approach to be effective at identifying a state set that corresponds well to clinically significant tumor types and at inferring phylogenetic relationships among them that are generally well supported by current knowledge about the molecular genetics of lung cancers.

## Results

### Algorithms

#### Model and definitions

We assume that the input to our methods consists primarily of a set of gene expression values describing activity of *d *genes in *n *tumor samples. These data are collectively encoded as a *d *× *n *gene expression matrix *M*, in which each column corresponds to expression of one tumor sample and each row to a single gene in that sample. We make no assumptions about whether the sample is representative of the whole patient population or biased in some unspecified way, although we would expect the methods to be more effective in separating states that constitute a sufficiently large fraction of all cells sampled across the patient population. The fraction of cells needed to give sufficiently large representation cannot be specified precisely, however, as it would be expected to depend on data quality, the number of components to be inferred, and the specific composition of each component. We define *m*_*ij *_to be element (*i*, *j*) of *M*. Note that it is assumed that *M *is a raw expression level, possibly normalized to a baseline, and not the more commonly used log expression level. This assumption is necessary because our mixing model assumes that each input expression vector is a linear combination of the expression vectors of its components, an assumption that is reasonable for raw data but not for logarithmic data. We further assume that we are given as input a desired number of mixture components, *k*. The algorithm proceeds in two phases: unmixing and phylogeny inference.

The output of the unmixing step is assumed to consist of a set of mixture components, representing the inferred cell types from the microarray data, and a set of mixture fractions, describing the amount of each observed tumor sample attributed to each mixture component. Mixture components, then, represent the presumed expression signatures of the fundamental cell types of which the tumors are composed. Mixture fractions represent the amount of each cell type inferred to be present in each sample. The degree to which different components co-occur in common tumors according to these mixture fractions provides the data we will subsequently use to infer phylogenetic relationships between the components. The mixture components are encoded in a *d *×*k *matrix *C*, in which each column corresponds to one of the *k *components to be inferred and each row corresponds to the expression level of a single gene in that component. The mixture fractions are encoded in an *n *× *k *matrix *F*, in which each row corresponds to the observed mixture fractions of one observed tumor sample and each column corresponds to the amount of a single component attributed to all tumor samples. We define *f*_*ij *_to be the fraction of component *j *assigned to tumor sample *i *and  to be vector of all mixture fractions assigned to a given tumor sample *i*. We assume that ∑_*i *_*f*_*ij *_= 1 for all *j*. The overall task of the unmixing step, then, is to infer *C *and *F *given *M *and *k*.

**Figure 1:**
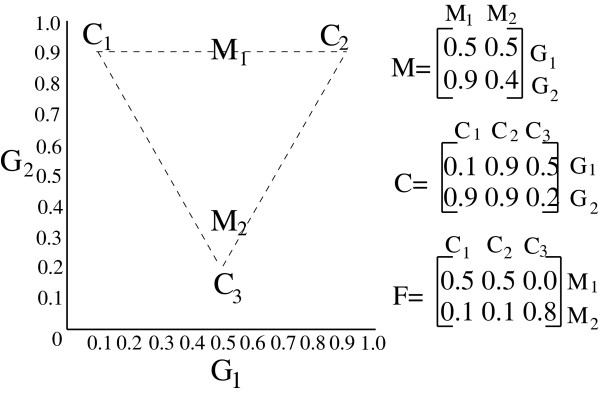
**Illustration of the geometric mixture model used in the present work**. The image shows a hypothetical set of three mixture components (*C*_1_, *C*_2_, and *C*_3_) and two mixed samples (*M*_1 _and *M*_2_) produced from different mixtures of those components. The triangular simplex enclosed by the mixture components is shown with dashed lines. To the right are the matrices *M*, *C*, and *F *corresponding to the example data points.

The unmixing problem is illustrated in Fig. 1, which shows a small hypothetical example of a possible *M, C*, and *F *for *k *= 3. In the example, we see two data points, *M*_1 _and *M*_2_, meant to represent primary tumor samples derived from three mixture components, *C*_1_, *C*_2_, and *C*_3_. For this example, we assume data are assayed on just two genes, *G*_1 _and *G*_2_. The matrix *M *provides the coordinates of the observed mixed samples, *M*_1 _and *M*_2_, in terms of the gene expression levels *G*_1 _and *G*_2_. We assume here that *M*_1 _and *M*_2 _are mixtures of the three components, *C*_1_, *C*_2_, and *C*_3_, meaning that they will lie in the triangular simplex that has the components as its vertices. The matrix *C *provides the coordinates of the three components in terms of *G*_1 _and *G*_2_. The matrix *F *then describes how *M*_1 _and *M*_2 _are generated from *C*. The first row of *F *indicates that *M*_1 _is a mixture of equal parts of *C*_1 _and *C*_2_, and thus appears at the midpoint of the line between those two components. The second row of *F *indicates that *M*_2 _is a mixture of 80% *C*_3 _with 10% each *C*_1 _and *C*_2_, thus appearing internal to the simplex but close to *C*_3_. In the real problem, we get to observe only *M *and must therefore infer the *C *and *F *matrices likely to have generated the observed *M*.

The output of the phylogeny step is presumed to be a tree whose nodes correspond to the mixture components inferred in the unmixing step. The tree is intended to describe likely ancestry relationships among the components and thus to represent a hypothesis about how cell lineages within the tumors collectively progress between the inferred cell states. We assume for the purposes of this model that the evidence from which we will infer a tree is the sharing of cell states in individual tumors, as in prior combinatorial models of the oncogenetic tree problem [11-13]. For example, suppose we have inferred mixture components *C*_1_, *C*_2_, and *C*_3 _from a sample of tumors and, further, have inferred that one tumor is composed of component *C*_1 _alone, another of components *C*_1 _and *C*_2_, and another of components *C*_1 _and *C*_3_. Then we could infer that *C*_1 _is the parent state of *C*_2 _and *C*_3 _based on the fact that the presence of *C*_2 _or *C*_3 _implies that of *C*_1 _but not vice-versa. This purely logical model of the problem cannot be used directly on unmixed data because imprecision in the mixture assignments will lead to every tumor being assigned some non-zero fraction of every component. We therefore need to optimize over possible ancestry assignments using a probability model that captures this general intuition but allows for noisy assignments of components. This model is described in detail under the subsection "Phylogeny" below.

#### Cell type identification by unmixing

We perform cell type identification by seeking the most tightly fitting bounding simplex enclosing the observed point set, assuming that this minimum-volume bounding simplex provides the most plausible explanation of the observed data as convex combinations of mixture components. Our method is inspired by that of Ehrlich and Full [30], who proposed this geometric interpretation of the unmixing problem in the context of interpreting geological data to identify origins of sediment deposits based on their chemical compositions. Their method proceeds from the notion that one can treat a set of mixture components as points in a Euclidean space, with each coordinate of a given component specified by its concentration of a single chemical species. Any mixture of a subset of these samples will then yield a point in the space that is linearly interpolated between its source components, with its proximity to each component proportional to amount of that component present in the sample. Interpreted geometrically, the model implies that the set of all possible mixtures of a set of components will define a simplex whose vertices are the source components. In principal, if one can find the simplex then one can determine the compositions of the components based on the locations of the vertices in the space. One can also determine the amount of each component present in each mixed sample based on the proximity of that sample's point to each simplex vertex. Ehrlich and Full proposed as an objective function to seek the minimum-size simplex enclosing all of the observed points. In the limit of low noise and dense, uniform sampling, this minimum-volume bounding simplex would exactly correspond to the true simplex from which points are sampled. While that model might break down for more realistic assumptions of sparsely sampled, noisy data, it would be expected to provide a good fit if the sample is sufficiently accurate and sufficiently dense as to provide reasonable support for the faces or vertices of the simplex. There is no known sub-exponential time algorithm to find a minimum-volume bounding simplex for a set of points and Erhlich and Full therefore proposed a heuristic method that operates by guessing a candidate simplex within the point set and iteratively expanding the boundaries of the candidate simplex until they enclose the full point set.

We adopt a similar high-level approach of sampling candidate simplices and iteratively expanding boundaries to generate possible component sets. There are, however, some important complications raised by gene expression data, especially with regard to its relatively high dimension, that lead to substantial changes in the details of how our method works. While the raw data has a high literal dimension, though, the hypothesis behind our method is that the data has a low intrinsic dimension, essentially equivalent to the number of distinct cell states well represented in the tumor samples. To allow us to adapt the geometric approach to unmixing to these assumed data characteristics, our overall method proceeds in three phases: an initial dimensionality reduction step, the identification of components through simplex-fitting as in Ehrlich and Full, and assignment of likely mixture fractions in individual samples using the inferred simplex.

For ease of computation, we begin our calculations by transforming the data into dimension *k *- 1 (i.e., the true dimension of a *k*-vertex simplex). For this purpose, we use principal components analysis (PCA) [25], which decomposes the input matrix *M *into a set of orthogonal basis vectors of maximum variance, and then use the *k *- 1 components of highest variance. This operation has the effect of transforming the *d *× *n *expression matrix *M *into a linear combination *PV *+ *A*, where *V *is the matrix of principal components of *M*, *P *is the weighting of the first *k *- 1 components of *V *in each tumor sample, and *A *is a *d *× *n *matrix in which each element *a*_*ij *_contains the mean expression level of gene *d *across all *n *tumor samples. The matrix *P *then represents a maximum variance encoding of *M *into dimension *k *- 1. *P *serves as the principal input to the remainder of the algorithm, with *V *and *A *used in post-processing to reconstruct the inferred expression vectors of the components in the original dimension *d*.

Note that although PCA is itself a form of unmixing method, it would not by itself be an effective method for identifying cell states. We would not in general expect cell types to yield approximately orthogonal vectors since distinct cell types are likely to share many modules of co-regulated genes, and thus similar expression vectors, particularly along a single evolutionary lineage. Furthermore, the limits of expression along each principal component are not sufficient information to identify the cell type mixture components, each of which would be expected to take on some portion of the expression signature of several components. For the same reasons, we would not be able to solve the present problem by any of the other common dimension-reduction methods similar to PCA, such as independent components analysis (ICA) [34], kernel versions of PCA or ICA [35], or various related methods for performing non-linear dimensionality reduction while preserving local geometric structure [36-38]. One might employ ICA or other similar methods in place of PCA for dimensionality reduction in the preliminary step of this method. However, since our goal is only to produce a low-dimensional embedding of the data, there is some mathematical convenience to deriving an orthogonal basis set with exactly *k *dimensions, something that is not guaranteed for the common alternatives to PCA. It is also of practical value in solving the simplex-fitting problem to avoid using dimensions with very little variance, an objective PCA will accomplish.

Once we have transformed the input matrix *M *into the reduced-dimension matrix *P*, the core of the algorithm then proceeds to identify mixture components from *P*. For this purpose, we seek a minimum-volume polytope with *k *vertices enclosing the point set of *P*. The vertices will represent the *k *mixture components to be inferred. Intuitively, we might propose that the most plausible set of components to explain a given data set is the most similar set of components such that every observed point is explainable as a mixture of those components. Seeking a minimum volume polytope provides a mathematical model of this general intuition for how one might define the most plausible solution to the problem. The minimum volume polytope can also be considered a form of parsimony model for the observed data, providing a set of components that can explain all observed data points while minimizing the amount of empty space in the simplex, in which data points could be, but are not, observed.

Component inference begins by chosing a candidate point set that will represent an initial guess as to the vertices of the polytope. We select these candidate points from within the set of observed data points in *P*. We use a heuristic biased sampling procedure designed to favor points far from one another, and thus likely to enclose a large fraction of the data points. The method first samples among all pairs of observed data points (*i*, *j*) weighted by the distance between the points raised to the *k*^*th *^power: || - ||^*k*^. It then successively adds additional points to a growing set of candidate vertices. Sampling of each successive point is again weighted by the volume of the simplex defined by the new candidate point and the previously selected vertices raised to the *k*^*th *^power. Simplex volume is determined using the Matlab convhulln routine. The process of candidate point generation terminates when all *k *candidate vertices have been selected, yielding a guess as to the simplex vertices that we will call *K*, which will in general bound only a subset of the point set of *P*.

The next step of the algorithm uses an approach based on that of Ehrlich and Full [30] to move faces of the simplex outward from the point set until all observed data points in *P *are enclosed in the simplex. This step begins by measuring the distance from each observed point to each face of the simplex. A face is defined by any *k *- 1 of the *k *candidate vertices, so we can refer to face *f*_*i *_as the face defined by *K*/{*k*_*i*_}. This distance is assigned a sign based on whether the observed point is on the same side of the face as the missing candidate vertex (negative sign) or the opposite side of the face (positive sign). The method then identifies the largest positive distance from among all faces *f*_*i *_and observed points *p*_*j*_, which we will call *d*_*ij*_. *d*_*ij *_represents distance of the point farthest from the simplex. We then transform *K *to enclose *p*_*j *_by translating all points in *K*/{*k*_*i*_} by distance *d*_*ij *_along the tangent to *f*_*i*_, creating a larger simplex *K *that now encloses *p*_*j*_. This process of simplex expansion repeats until all observed points are within the simplex defined by *K*. This final simplex represents the output of one trial of the algorithm. We repeat the method for *n *trials, selecting the simplex of minimum volume among all trials, *K*_*min*_, as the output of the component inference algorithm.

Once we have selected *K*_*min*_, we must explain all elements of *M *as convex combinations of the vertices of *K*_*min*_. We can find the best-fit matrix of mixture fractions *F *by solving for a linear system expressing each point as a combination of the mixture components in the *k *- 1-dimensional subspace. To find the relative contributions of the mixture components to a given tumor sample, we establish a set of constraints declaring that for each gene *i *and tumor sample *t*:

We also require that the mixture components sum to one for each tumor sample:

Since there are generally many more genes than tumor samples, the resulting system of equations will usually be overdetermined, although solvable assuming exact arithmetic. We find a least-squares solution to the system, however, to control for any arithmetic errors that would render the system unsolvable. The *f*_*tj *_values optimally satisfying the constraints then define the mixture fraction matrix *F*.

We must also transform our set of components *K*_*min *_back from the reduced dimension into the space of gene expressions. We can perform that transformation using the matrices *V *and *A *produced by PCA as follows:

The resulting mixture components *C *and mixture fractions *F *are the primary outputs of the code. The full inference process is summarized in the following pseudocode:

Given tumor samples *M *and desired number of mixture components *k*:

1. Define *K*_*min *_to be an arbitrary simplex of infinite volume

2. Apply PCA to yield the *k *- 1-dimension approximation *M *≈ *PV *+ *A*

3. For each *i *= 1 to *n*

a. Sample two points  and  from P weighted by || - ||^*k*^

b. For each *j *= 3 to *k*

i. Sample a point  from *P *weighted by *volume*(, ..., )^*k*^

c. While there exists some *p*_*j *_in *P *not enclosed by *K *= (, ..., )

i. Identify the *p*_*j *_farthest from the simplex defined by *K*

ii. Identify the face *f*_*i *_violated by *p*_*j*_

iii. Move the vertices of *f*_*i *_along the tangent to *f*_*i *_until they enclose *p*_*j*_

d. If *volume*(*K*) <*volume*(*K*_*min*_) then *K*_*min *_← *K*

4. For each tumor sample *i*

i. Solve for the elements *f*_*tj *_of *F *defined by the constraints:

∑_*j *_*f*_*tj*_*k*_*ij *_= *p*_*it *_∀*i*, *t*

∑_*j *_*f*_*tj *_= 1 ∀ *t*

5. Find the component matrix *C *← *K*_*min*_*V *+ *A*

6. Return (*C*, *F*) as the inferred components and mixture fractions

#### Phylogeny inference

Once we have inferred cell states and their mixture fractions in each tumor sample, we can use those inferences to construct a phylogeny suggesting how the states are evolutionarily related. The sharing of states within individual tumors provides clues as to which cell types are likely to occur on common progression pathways. Imprecision in the mixture fraction assignments, however, will tend to create a spurious appearance of cell-type sharing due to tumors being assigned some non-zero fraction of each cell type whether or not they truly contain that type. To overcome the confounding effects of this noise in the mixture fractions, we pose phylogeny inference as the problem of finding a tree that maximizes cell-type sharing across tree edges and thus implicitly minimizes the assignment of edges to cell-type pairs that appear to co-occur due to noisy mixture fraction assignments or more distant evolutionary relationships. We define a measure of sharing of any two cell types *i*, *j *as follows:

where *t *sums over tumor samples.

One can conceive of this measure as a log likelihood model, in which we are interested in explaining the frequency with which any given pair of states would be sampled by picking two independent cells from a given tumor. The numerator describes the hypothesis that a given pair of states are sampled from correlated densities, with the frequency of the pair derived by summing over the product of the two types' frequencies in individual tumors. The denominator describes the hypothesis that the states are independent of one another and thus sampled independently from some background noise distributions, with the two independent frequencies estimated by summing each cell type's frequency individually over all tumors. Seeking a tree that maximizes the log sum of this measure across all tree edges is then equivalent to seeking a maximum likelihood Bayesian model in which each child is presumed to have frequency directly dependent on its parent and independent of all other tree nodes. Intuitively, this distance function will tend to assign high sharing to cell types that generally have high frequencies in common tumors and low sharing to cell types that generally occur in disjoint tumors. The set of *s*_*ij *_values thus provides a similarity matrix for a phylogeny inference.

The model makes several assumptions about the available data. We assume that we have inferred all states present in the data and that our states therefore represent both internal and leaf nodes of the phylogeny. This assumption follows from the evidence that tumor samples maintain remnant populations of their earlier progression states [17-19], leading to the conclusion that our model should be able to explain some states as ancestors of others. While it is possible that some ancestral states are lost or preserved at levels too low to detect, we do not attempt to infer the presence of missing (Steiner) states. We further assume that the evolutionary relationships among the states are in fact a tree, i.e., connected and cycle-free. Finally, we assume that all observed states are in fact related to one another. It is indeed possible that any of these assumptions could be violated. Our prior work on phylogenetics from single-cell fluoresence in situ hybridization (FISH) data suggests that there may be multiple pathways from healthy cells to particular tumor states [15,16], which would imply that the true evolutionary pathways may form a cycle-containing phylogenetic network rather than a phylogenetic tree. It is also reasonable to suppose that different tumors may originate from distinct cell types and thus form a multi-tree forest, rather than a single tree. For the present proof-of-concept study, though, we have chosen to exclude these possibilities in order to avoid the greater uncertainty we would incur by seeking to fit to a richer class of models. Furthermore, we would expect that tumor samples will contain contamination from stromal cells that might not be ancestral to any of the tumor cells. We again choose not to build in an explicit correction to our model to distinguish tumor from healthy cells in our model. Rather, we allow the model to treat contaminating healthy cells as one or more tumor states, expecting that healthy cells in the mixtures will be inferred as ancestral states to the tumor whether or not the tumor actually arose from the same population of healthy cells as those it has infiltrated. Thus, we model our phylogeny problem strictly as the problem of inferring a maximum-similarity tree connecting all of our observed states without the introduction of additional (Steiner) nodes. For this model, we can pose the problem as a minimum spanning tree (MST) problem in which each edge (*i*, *j*) is assigned weight -*s*(*i*, *j*). We solve this problem with the Matlab graphminspantree routine.

### Testing

#### Validation on simulated data

We first validated the method using two protocols for simulated data generation. Simulated data is essential for validation because the ground truth components and their representation in particular tumors are not known for real tumor data sets. In addition, it allows us to explore how performance of the method varies with assumptions about the data set. We began by applying a simple simulation protocol for generating uniformly sampled mixtures, in which each component is simulated as an independent vector of unit normal random variables and each observed tumor passed as input to the data set is simulated as a uniformly random mixture of this common set of components (see Methods). We developed a second simulation protocol meant to better mimic the substructure expected from true tumor samples due to the evolutionary relationships among sub-types. In this protocol, we assume that mixture components correspond to nodes in a binary tree and that each observed tumor represents a mixture of components along a random path in that tree (see Methods). In both protocols, we add log normal noise to all simulated expression measurements.

**Figure 2:**
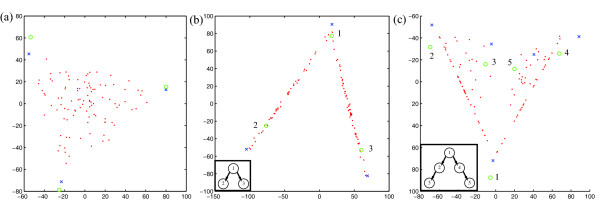
**Examples of mixture components inferred from simulated data sets**. Green circles show the true mixture components, red points the simulated data points that serve as the input to the algorithms, and blue X's the inferred mixture components. (a) A uniform mixture of three independent components with no noise. Each data point is a mixture of all three components. Inferred mixture fractions for the three components, averaged over all points, are (0.295 0.367 0.339). (b) A tree-embedded mixture of three components with noise equal to signal. Each data point is a mixture of a root component (top, labeled 1) and one of two leaf components (bottom, labeled 2 and 3). The inset shows the phylogenetic tree in which the labeled components are embedded. Inferred mixture fractions averaged over points in the two branches of the simplex are (0.410 0.567 0.025) and (0.410 0.020 0.535) (c) A tree-embedded mixture of five components with 10% noise. Each data point contains a portion of the root component (bottom, labeled 1), a subset contain portions of one of two internal components (far left, labeled 2, and far right, labeled 4), and subsets of these contain portions of one of two leaf components (center left, labeled 3, and center right, labeled 5). The inset shows the phylogenetic tree in which the labeled components are embedded. Inferred mixture fractions averaged over points in the two branches of the simplex are (0.356 0.462 0.141 0.006 0.005) and (0.387 0.072 0.008 0.187 0.378).

Fig. 2 shows a few illustrative examples of simulated data sets along with their true and inferred mixture components. Fig. 2(a) shows a trivial case of the problem, a uniform mixture of three components without noise, resulting in a triangular point cloud. The close overlap of the true mixture components (circles) and the inferred components (X's) shows that method could infer the mixture components in this case with high accuracy. Fig. 2(b) shows a tree-embedded sample of three components in the presence of high noise (signal equal to noise). Performance was somewhat degraded, apparently primarily because the simplex produced by the true mixture components was a poorer fit to the noisy data. Fig. 2(c) shows a more complicated evolutionary scenario consisting of five tree-embedded mixture components, with low (10%) noise. The scenario models two progression lineages, with each sample consisting of a component of the root state and zero, one, or two states along a single progression lineage. The result is a simplicial complex consisting of two triangular faces joined at the root point. While there was a clear correspondence between true and inferred mixture components, performance quality was noticeably lower than that for the simpler scenarios.

**Figure 3:**
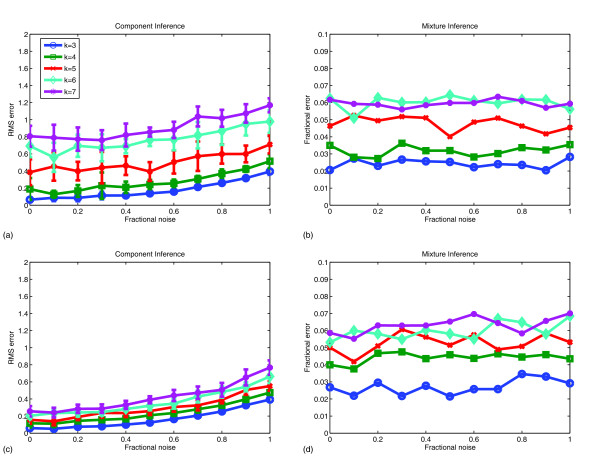
**Accuracy of methods in inferring simulated mixture components and assigning mixture fractions to data points**. (a) Root mean square error in inferred mixture components as a function of noise level for uniform mixtures of *k *= 3 to *k *= 7 mixture components. (b) Root mean square error in fractional assignments of components to data points as a function of noise level for uniform mixtures of *k *= 3 to *k *= 7 mixture components. (c) Root mean square error in inferred mixture components as a function of noise level for tree-embedded mixtures of *k *= 3 to *k *= 7 mixture components. (d) Root mean square error in fractional assignments of components to data points as a function of noise level for tree-embedded mixtures of *k *= 3 to *k *= 7 mixture components.

Fig. 3 quantifies the performance quality across a range of simulated data qualities and evolution scenarios. Fig. 3(a) assesses accuracy on uniform mixtures by the error in inferred components and Fig. 3(b) by the error in inferred mixture fractions. Figs. 3(a, b) reveal that mixture components could be identified with high accuracy provided there were few mixture components and low noise. Accuracy degraded as component number or noise level increased. Errors appear to have grown superlinearly with component number but sublinearly with the noise level. Accuracy of mixture fraction inference appears sensitive to component number but largely insensitive to noise level over the ranges examined here. It should be noted that the high accuracy regardless of noise level likely depended on the assumption that noise in each gene is independent, allowing extremely accurate estimates when noise could be averaged over many genes. Correlated noise between genes or systemic sample-wide errors would be expected to yield poorer performance.

Figs. 3(c, d) provide a comparable analysis for tree-embedded samples. The tree-embedded data yielded qualitatively similar trends to the uniform mixtures. Component inference degraded with increasing noise or increasing number of components while mixture fraction inference degraded with increasing number of components but appears insensitive to noise level. Compared to uniform samples, tree-embedded samples led to substantially better inference of components but generally slightly worse inference of mixture fractions.

**Figure 4:**
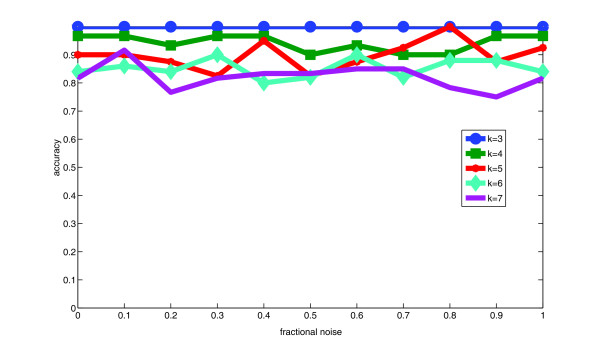
**Accuracy of tree inference on simulated tree-embedded data**. The plot shows the fraction of true tree edges accurately inferred for *k *= 3 to *k *= 7 components as functions of noise levels.

Fig. 4 plots accuracy of tree inference on tree-embedded simulated data, measured as the fraction of true tree edges correctly inferred over ten replicates per data point. Accuracy ranged from 100% for three-component inferences to approximately 75%-80% for seven-component inferences. Accuracy appears to have been insensitive to noise in expression measurements over the ranges examined. The fraction of edges one would expect to correctly predict by chance for a *k*-node tree is (*k *- 1)/, which ranges from 67% for *k *= 3 to 29% for *k *= 7. We can thus conclude that the performance, while not perfect, was substantially better than would be observed by chance.

#### Application to real data

In order to demonstrate the applicability of the methods to real tumor data, we next examined a dataset of lung tumor expression measurements from Jones et at. [33]. This dataset is particularly useful for the present validation because it includes normal lung samples, which allow us to root phylogenies and look for expected mixing of normal cells in tumor samples; because it is well annotated with regard to clinically significant tumor subtypes, which provides a partial basis for validating the success of the unmixing; and because it includes both primary tumor samples and cell lines for a single tumor type, which allows us to compare inferred mixture fractions between "pure" and "mixed" samples. The authors of this study classified tumors into one of eight categories: normal lung cells (19 samples), primary adenocarcinoma (12 samples), primary large cell carcinoma (12 samples), primary carcinoid (12 samples), primary small cell (15 samples), small cell lines (11 samples), primary large cell neuroendocrine (8 samples), and primary combined small cell/adenocarcinoma (2 samples). These categories are used for validation and visualization purposes below.

**Figure 5:**
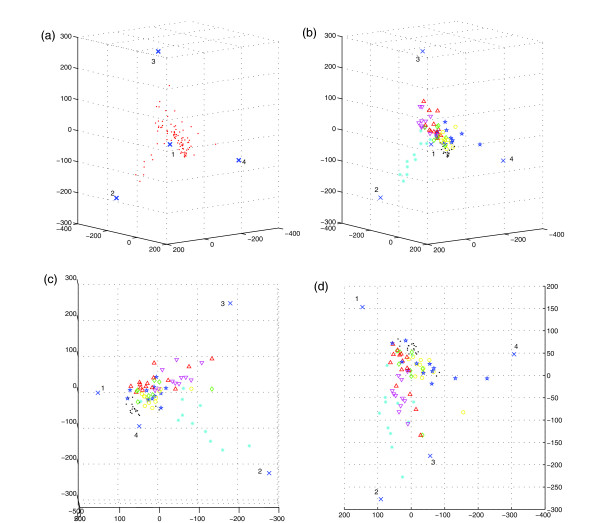
**Visualization of four-component unmixing results from the lung cancer data of Jones et al. **[33]. (a) All components and tumor samples. Tumor samples appear as red points and components as blue X's labeled by numbers. (b-d) Three views of the same data with distinct clinical subtypes highlighted. Components appear as blue X's labeled by numbers. Tumors are marked as follows: normal lung tissue (black point), large cell carcinoma (blue star), carcinoid (cyan asterisk), adenocarcinoma (yellow circle), large cell neuroendocrine (green diamond), small cell primary tumors (red upward-pointing triangles), small cell cell lines (magenta downward-pointing triangles). The two primary combined small cell/adenocarcinoma samples were omitted from (b-d).

Fig. 5 visualizes the results of the four-component inference on the Jones et al. data [33]. Fig. 5(a) shows the full set of data points, each visualized as a red point, and the set of mixture components, shown as blue X's. The positions of the mixture components in relative gene expression space are provided in Additional file 1, Table S1. We have added numerical labels (1-4) to the inferred mixture components to allow unambiguous reference to them below. We will subsequently refer to these four inferred mixture components as , , , and . While the three-dimensional fit of the data points into the simplex is difficult to visualize from two-dimensional projections, it can be roughly described as a dense central point cloud from which three "arms" project to form a tripod shape. Mixture component  was placed above the central cloud on the opposite side from the arms and , , and  each fell roughly along the vector of a distinct arm, somewhat beyond that arm's end.

Fig. 5(b-d) provides three additional views with the individual tumors marked to indicate clinical subtypes. Fig. 5(b) shows a view of the three "arms" seen from above the central cloud. Normal lung cells (black points) clustered near the top of the central cloud, with adenocarcinoma (yellow circles) and large-cell neuroendocrine tumors (green diamonds) nearby.  appears near the middle of the figure in this view, near the central cloud but above and somewhat off-center. The first arm extends to the lower left towards  and appears to consist exclusively of carcinoid tumors. The second arm extends upward towards  and consists primarily of small cell lung cancers, both primary and cell line. A third arm, apparently consisting primarily of large cell carcinomas, extends towards . Fig. 5(c) shows an alternative view approximately down the axis running from  to the central cloud. This view makes it more apparent that  was positioned just beyond the central cloud and its cap of normal cells, although somewhat skewed towards the small cell tumors. This view also reveals that large cell neuroendocrine tumors lie between normal and small cell tumors and that small cell lines lie further towards  than do small cell primary samples. Fig. 5(d) provides one additional view, meant to highlight the large cell axis towards . In this view, adenocarcinomas appear to lie along the vector from normal cells to large cell carcinomas. On the basis of these observations, we could approximately associate the four components with the clinical subclasses as follows:  with normal cells,  with carcinoid,  with small cell and large cell neuroendocrine, and  with large cell carcinoma and adenocarcinoma.

Table 1 shows average fractional assignments of components to each of the tumor types, allowing us to quantify these visual impressions. The table confirms the association of clinical subtypes with components suggested in the preceding paragraph, although with significant noise.  was the predominant component of normal cells, although it was also well represented in most other tumor types. Small cell primary tumor samples showed a larger fraction of the normal component than do small cell lines, as we would expect, although the small cell lines still showed a high representation of the normal component.  shows strong specificity specifically for carcinoid tumors.  most specifically marked the combined small cell/adenocarcinomas, followed by small cell lines, small cell primary tumors, and large cell neuroendocrine.  marked combined small cell/adenocarcinoma, adenocarcinoma, and large cell carcinoma. All components, however, showed non-specific representations of approximately 5-20% across tumor types, suggestive of an insufficiently tight fit of the simplex to the data.

**Table 1:** Mixture fractions averaged by tumor type for a four component inference.

	Comp. 1	Comp. 2	Comp. 3	Comp. 4
Normal	0.5387	0.1165	0.0451	0.2998

Adenocarcinoma	0.3943	0.1196	0.1708	0.3153

Small cell (cell lines)	0.4074	0.1701	0.3493	0.0732

Small cell (primary)	0.5015	0.0952	0.2665	0.1368

Carcinoid	0.3431	0.4198	0.1304	0.1066

Large cell neuroendocrine	0.4674	0.1227	0.2093	0.2007

Large cell carcinoma	0.4010	0.0836	0.1861	0.3293

Combined SCLC/AD	0.0650	0.1586	0.4120	0.3643

Columns correspond to mixture components from a four-component inference. Rows correspond to distinct tumor types, as annotated by Jones *et al*. [[33]].

We next examined performance of the method with six components, which we label , ..., . While we cannot easily visualize the resulting five-dimensional simplex, we can interpret the results in terms of mixture fraction assignments (Table 2) and gene expression levels assigned to the components (Additional file 2, Table S2). Table 2 suggests that  predominantly marks the normal cells (analogous to ); that  predominantly marked the large cell carcinoma, adenocarcinoma, and combined small cell/adenocarcinoma (analogous to ); that  predominantly marked the small cell lines, followed by small cell primary tumors and neuroendocrine tumors (analogous to ); and that  was specific for the carcinoid tumors (analogous to ).  did not have an easy interpretation in terms of tumor types, as it most strongly marked the small cell cancers but was strongly associated with normals and assigned as a high fraction for all but the combined tumors.  showed high frequency only for combined small cell/adenocarcinomas; in fact, a closer examination of the data showed it to be strongly associated with only one of the two combined tumors (56.8%) and only minimally with the other (16.6%).

**Table 2:** Mixture fractions averaged by tumor type for a six component inference.

	Comp. 1	Comp. 2	Comp. 3	Comp. 4	Comp. 5	Comp. 6
Normal	0.4017	0.1134	0.0993	0.0563	0.2338	0.0955

Adenocarcinoma	0.2625	0.1748	0.0960	0.0627	0.2556	0.1483

Small cell (cell lines)	0.1585	0.0705	0.2818	0.0645	0.3151	0.1095

Small cell (primary)	0.2539	0.0677	0.1913	0.0301	0.3252	0.1317

Carcinoid	0.1670	0.0674	0.1681	0.2868	0.2358	0.0747

Large cell neuroendocrine	0.2591	0.1206	0.1955	0.0434	0.2977	0.0838

Large cell carcinoma	0.2461	0.2110	0.1264	0.0257	0.2852	0.1056

Combined SCLC/AD	0.1270	0.2000	0.1425	0.0695	0.0938	0.3672

Columns correspond to mixture components from a six-component inference. Rows correspond to distinct tumor types, as annotated by Jones *et al*. [[33]].

Comparing gene expression vectors from Additional files 1 and 2, Tables S1 and S2, can help us interpret these observations. Table 3 shows Pearson correlation coefficients between the inferred expression vectors of the two component sets. The table suggests that  has been largely captured by  and  by .  has been split into contributions of  and .  was split into contributions of , , and .

**Table 3:** Correlations between four- and six-component inferences by inferred gene expression vectors.

	Comp. 1	Comp. 2	Comp. 3	Comp. 4	Comp. 5	Comp. 6
Comp. 1	0.3348	-0.5376	-0.1510	-0.2595	0.3040	-0.3127

Comp. 2	-0.1271	-0.1618	0.2200	0.9450	-0.1983	-0.1663

Comp. 3	-0.6752	-0.2433	0.4800	-0.3663	0.2977	0.5466

Comp. 4	0.3515	0.8621	-0.4429	-0.2310	-0.3799	-0.0187

Entries show the Pearson correlation coefficients between inferred relative gene expression levels for components inferred from a 4-component versus a 6-component inference. Rows correspond to mixture components from a four component inference and columns from a six component inference.

**Figure 6:**
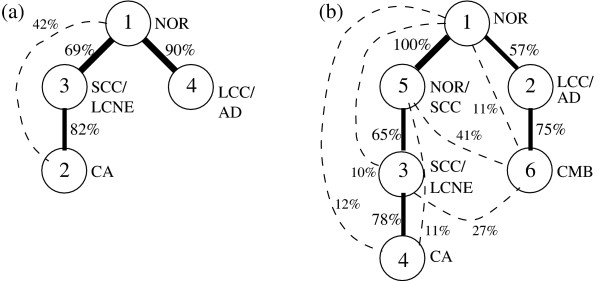
**Phylogenies inferred on components derived from Jones et al**. [33]. Each phylogeny shows nodes labeled with component numbers. We further manually added labels reflecting approximately which tumor types are most specifically labeled by a given component based on Tables 1 and 2: NOR (normal cells); LCC/AD (large cell carcinoma and adenocarcinoma); SCC (small cell); CA (carcinoid); CMB (combined small cell/adenocarcinoma) and NOR/SCC (normal and small cell). Edges with over 50% confidence are shown as solid lines while those between 10% and 50% confidence are shown as dashed lines. Edges with confidence below 10% are omitted. Edges are labeled by confidences rounded to the nearest percent. (a) Phylogeny derived from four mixture components. (b) Phylogeny derived from six mixture components.

Fig. 6 shows the phylogeny inferences derived from the lung cancer data. For ease in interpretation, we have labeled nodes of the trees by the tumor types with which they are most strongly associated. The 4-component phylogeny (Fig. 6(a)) showed lung tumors following two distinct pathways from normal cells. The first pathway moved first towards a small cell/large cell neuroendocrine state. Carcinoid tumors appeared as a later branch off that first state. There was, however, uncertainty about the order of those two states, with a substantial fraction of trials placing carcinoid above small cell/large cell neuroendocrine or placing the two states as independent branches off normal cells. The second pathway led into the large cell carcinoma/adenocarcinoma state. The 6-component phylogeny (Fig. 6(b)) likewise showed two major pathways, one passing through small cell/large cell neuroendocrine and terminating in carcinoid tumors, the other passing through large cell carcinoma/adenocarcinoma. If we use the correspondence of states suggested by tumor type associations ( to ,  to ,  to , and  to ) then the 4-component tree could be seen to be a subgraph of the 6-component tree. One of the two additional states () was inserted as an early step along the small cell/large cell neuroendocrine/carcinoid pathway. The other (), which showed preference for the combined SCC/AD tumors, appeared as a later state along the large cell carcinoma/adenocarcinoma pathway. The greatest uncertainty in the tree concerned the placement of , which was most often a child of  but was found with moderate probability as children of the three normal and small cell states. The 6-component tree, like the 4-component tree, also showed uncertainty in the placement of the carcinoid state, which was most often placed as a descendant of the small cell/neuroendocrine states but was often placed higher in that lineage or in a separate lineage out of the normal cells.

The application to real lung cancer data provides a further opportunity to evaluate the approach, in addition to suggesting some novel hypotheses about the molecular evolution of lung cancers. Both 4- and 6-component inferences suggest that the tumor types examined here evolve into two major groups early in their progression: one consisting of large cell neuroendocrine, small cell, and carcinoid tumors and the other consisting of adenocarcinoma and large cell carcinoma. This subdivision is supported by many lines of evidence on specific genetic abnormalities frequently found in the different sub-types, which suggest that small cell and large cell neuroendocrine carcinomas arise from common progenitors [39] and that carcinoids are closely related to neuroendocrine and small cell tumors but not to other carcinomas [40,41]. This conclusion thus appears to provide validation for the method. Both trees also suggest that carcinoid cells represent a later stage of progression than large cell neuroendocrine and small cell tumors along a common lineage. While the literature does support a close relationship between these sub-types, it also suggests that carcinoid tumors likely represent a less advanced state of progression than small cell tumors. Several genetic abnormalities characteristic of both types show higher frequency in small cell than in carcinoid tumors [42,43]. Furthermore, small cell tumors are more aggressive than carcinoids [44]. It thus appears that while the close placement of these states provides further validation for the present approach, it has most likely inverted the order of those states along the lineage. The fact that the method relies on sharing of states within common tumors to make phylogenetic inferences may make it particularly vulnerable to this sort of inversion of states along a single lineage absent higher-quality assignments of mixture fractions.

The 6-component tree appears to be an elaboration on the 4-component tree, supporting a common model of the two major pathways but adding some additional features beyond that. One intriguing feature is the insertion of the  component at the top of the neuroendocrine/small cell/carcinoid lineage. The method appears to have sub-divided the normal cells into two populations: a more specifically normal state () and a state more strongly shared with the tumors and especially the small cell lineage (). It is possible this split represents some axis of variation independent of mutational state of a cell. It could also, however, suggest the hypothesis that the separation into the two tumor lineages corresponds to a genetic or epigenetic heterogeneity present within normal lung cells. In particular, the phylogeny predicts that the small cell lineage specifically branches from  and the large cell lineage from . The other elaboration is the introduction of , which is most specifically associated with a single combined small cell/adenocarcinoma tumor. The existence of such combined tumors would appear incompatible with the conclusion that small cell and adenocarcinoma lie on unrelated lineages. Examination of molecular abnormalities in specific cases is contradictory on this point, though, with some studies reporting combined tumors that arise through the independent appearance of distinct cancer lineages in a single patient [45] but others showing evidence of common clonal origin [46]. The inferred phylogeny suggests that this new state is most likely a later progression of an adenocarcinoma state, with the combined tumors seemingly acquiring their small cell component independently. Given the high uncertainty of the phylogeny step in placing  in the tree, though, we might alternatively suggest that  is simply a spurious inference that cannot be confidently placed because it does not correspond to a true progression state of lung tumors. The two combined tumors included in the Jones et al. [33] dataset used in the present study show very different expression patterns and mixture assignments from one another. The geometric approach chosen here would be expected to be sensitive to outlier points and it may be that aberrant expression in even a single unusual tumor could have forced incorrect inference of a new progression state.

### Implementation

All code for this project was written in Matlab and executed with Matlab v.7 on a Linux PC. Matlab was also used for visualization of component inferences. The validation code also required Hungarian.m, a third-party Matlab routine for performing weighted bipartite maximum matching [47]. Unmixing and phylogenetics code implementing the algorithms described in the present work are available from

http://www.cs.cmu.edu/~russells/software/unmixing/

as Matlab ".m" files. Other custom code used in data analysis and generation of simulated data sets will be provided upon request.

## Discussion

We have developed a novel approach to tumor phylogenetics combining unmixing methods with a cell-by-cell strategy for phylogeny inference. The method is an attempt to gain the advantages of both intratumor heterogeneity information available to cell-by-cell methods and the large probe sets available to tumor-by-tumor methods. The application to simulated data sets suggests that the method is effective at making component and mixture fraction inferences from large, noisy datasets for limited numbers of components. The method does, however, degrade in performance quickly with increasing numbers of mixture components. Phylogeny inference, which depends on the quality of the mixture fraction inference, similarly shows high tolerance for noise, although a loss of quality with increasing numbers of components. Nonetheless, phylogeny inferences show good reconstruction accuracy for as many as seven components on simulated tree-embedded samples. The methods are generally more effective at component inference from these tree-embedded samples, suggesting that they can effectively exploit some features of the geometric substructure we would expect an evolutionary process to produce. Application to a real lung cancer data set shows the method to be effective at inferring components consistent with known lung cancer sub-types and with grouping these components into phylogenies generally consistent with the prior literature on the evolution of lung tumors. The method does, however, make some apparent mistakes and provide low confidence to some seemingly correct predictions, suggesting room for improvement.

The approach, at least as presently realized, does make a number of assumptions about the data and tumor progression in general that one might reasonably question. The primary purpose of this paper is to lay out the general concept of how unmixing methods can inform tumor phylogenetics and this concept in itself implies certain assumptions. Some assumptions concern the biology of tumor development: that there are reproducible cancer sub-types that co-occur in the population, that tumors accumulate remnant cell populations as they progress, that these remnant progression states are themselves reproducible across patients, and that these remnant states persist at high enough levels to measureably influence overall expression. There is considerable literature supporting all of these points, as discussed in the Introduction, although they might reasonably be debated. A further assumption is that the states we wish to observe differ sufficiently in expression profile as to be separable by unmixing methods. While there is strong evidence in the literature that distinct sub-types can be separated by their expression profiles, there is no direct experimental basis from which to argue that individual cell states along a single progression pathway are similarly separable. It remains to be seen how precisely one can sub-divide a progression pathway and which mutations or combinations of mutations will or will not lead to discernible changes in expression profile.

The specific implementation of the model in the present work adds additional assumptions that underlie the results here but might conceivably be relaxed in future work. The present model assumes that individual components of a mixture contribute linearly to the mixture, i.e., that the expression level of each gene in a tumor sample is a derived from the weighted sum of the expression level of the gene in each component of the sample. This assumption might break down due to limitations of the microarray technology or for biological reasons, e.g., if intracellular communication leads to radically different expression in mixtures of cell types than in the cell types independently. We would expect the dimensionality reduction step to partially correct for such problems by extracting a linear subset of the full expression space. Non-linear dimensionality reduction methods [36-38], however, might be more effective at making use of the available data if the linearity assumption is poorly satisfied. A second assumption of the present work is that we can unmix a sufficient number of states to produce a useful picture of progression. Any unmixing method will have difficulty separating rare states or states with very similar expression profiles, especially in the presence of noisy data. More sophisticated methods for noise-tolerant learning would seem to be a possible solution, such as the Gaussian mixture models used by Etzioni et al. [31]. Our current approach also assumes that we can do a reasonably good job of fitting a tight simplex to the point set. There is no known method sub-exponential in dimension for optimally solving the simplex-fitting problem and we must therefore use heuristics that provide no guarantees of the quality of our solutions. Nonetheless, improved simplex fitting might lead to a better ability to detect and reliably separate similar or rare states. Our current approach might be improved by post-processing with a local optimization method, such as the "simplex shrink-wrap" algorithm of Fuhrmann [48]. Another key step for improvement will be making better use of the geometric form of the data points expected. As both the lung data and the synthetic tree-embedded tumor data show, point sets derived from evolutionary processes are not uniform simplices, but rather more complicated simplicial complexes characterized by lower-dimensional elaborations branching from the ancestral states. Taking better advantage of this class of geometric structure provides a possible avenue for improving inference in the face of sparse and/or noisy data and avoiding the "curse of dimensionality" that leads to poor scaling in component number. Uncertainty in the predictions also suggests that more data may be needed to reliably learn the underlying structure. Even absent improvements in the methods, more or better data may lead to more nuanced and reliable predictions about tumor phylogenetics by this approach.

There are also several potentially debatable assumptions underlying our current phylogeny methods. The most obvious assumption is that all of the cell types we detect are in fact evolutionarily related. The ultimate product of our method will be a phylogeny connecting all of the inferred cell types, which will not in general be a meaningful outcome if the cell types are not related. At some level, of course, all cells in a given individual are related. Distinct tumors may, however, arise from different populations of healthy cells. Furthermore, stromal contamination will in general lead to tumor samples containing mixtures of healthy cells that may not be ancestral to any of the tumor types. We chose to accept this assumption in the model, even knowing it to be imperfect, in the belief that it is better to have the predictions of the method about ancestry available and account for the assumptions after the fact in interpreting the meaning of the inferred ancestry relationships. One might alternatively seek to explicitly separate healthy from cancerous cells prior to phylogenetic inference, using the mixture model to remove stromal contaminants as in Etzioni et al. [31]. A related assumption is that there are no missing states that need to be considered in the model. It is indeed possible that there are missing ancestral states that are present at too low a level to infer directly. In particular, if one accepts the cancer stem cell hypothesis [49,50] then it would be reasonable to infer that the observed states should all be leaf nodes of the phylogeny, with cancer stem cells comprising the internal nodes of the trees. One could adapt the method to that alternative model by replacing our minimum spanning tree step with any standard species tree phylogeny method, which would treat all observed samples as leaf nodes and all internal nodes as unobserved states. General Steiner tree inference methods might allow an intermediate solution, allowing for the assumption that observed states can be leaf or internal nodes but that additional internal nodes may be unobserved. A further assumption is that the output should be a tree. Prior studies on cell-by-cell data [15,16] show that single states may be reachable from multiple pathways, suggesting that a more general phylogenetic network model might be preferable. One final implicit assumption is that the only evidence available to us for phylogeny inference is the frequency with which states are shared in tumors, excluding information available to us in the expression vectors themselves. We might have alternatively posed the phylogeny inference step to operate on the assumption that cells with similar expression profiles are likely to be related, as was done by Desper et al. in examining expression profiles of whole tumors [13], or by combining the two sources of information.

A related question raised by these assumptions is whether we can tell, either in advance or post-hoc, whether the assumptions of the model are in fact satisfied by a given data set. One can in principal assess how well the raw input data is explained by a linear mixture of a small number of components, for example by examining the rate at which singular values of the matrix decay. Such a validation does not tell us whether information not accounted for by the linear model reflects genuinely non-linear contributions, the need for a more complex linear model, or noise in the true expression data or the experimental measurements. Neither does it tell us whether the linear model extracted carries sufficient information to characterize the cell states and their relationships. One can also apply a generic post-hoc validation based on how reproducible the results are to sub-sampling, as in our bootstrapping for the lung phylogenies. If the assumptions of the model are violated, then we would expect the data to only weakly support a defined tree topology. The lung cancer example suggests mixed success, with some features of the inferred trees strongly supported across subsamples but others nearly arbitrary. We can also, after the fact, examine the degree to which the inferred mixture fractions conform to the phylogeny. Healthy cells, when available, should be assigned minimal fractions of non-healthy components and tumors on a given progression pathway should exhibit minimal contamination with components characteristic of other pathways. While the lung data does show a clear partitioning of mixture components by sub-type, it nonetheless shows frequent contamination suggestive of poor fitting of the simplex. All of these measures suggest room for improvement in the model and algorithms and will provide a basis for determining whether any alternative methods do in fact improve on those proposed here. Perhaps the ultimate test of the method is how well it recapitulates what we already know about a given real data set. Our comparison of the lung cancer results to prior knowledge again suggests that the method works sufficiently well to recapitulate our prior understanding about the classification and origin of the major lung tumor classes, but with less precision than we might like and with some apparent mistakes. Further post-hoc validation might also be conducted directly on the derived components by testing whether they meet the expression signatures of known tumor sub-types or healthy cell populations.

## Conclusions

We have presented a novel method for identifying likely pathways of tumor progression using computational unmixing methods to interpret expression measurements from tumor samples as mixtures of fundamental components. Validation on simulated data demonstrates good effectiveness at inferring mixture components, assigning mixture fractions to samples, and inferring phylogenies provided noise levels and numbers of components are sufficiently small. The prototype methods presented here do appear to suffer from insufficiently precise fits of polytopes to the data, especially as the number of components increases, which can in turn result in spurious identification of components in samples that lack them and inaccurate phylogeny inferences. Nonetheless, the effectiveness of the models across a variety of scenarios and in the presence of relatively high levels of noise suggests that the approach has good promise for improving our ability to identify phylogenetic relationships among tumor cells. Likewise, application of the method to real lung cancer microarray data shows that the method to be effective at identifying components corresponding to known clinical sub-types and at inferring progression pathways largely consistent with current knowledge about the molecular evolution of lung tumors. These inferences also suggest some novel hypotheses about the genesis of lung cancers. The methods developed here thus represent a promising new computational model for phylogenetic studies of tumors that can provide many of the competing advantages of the two major paradigms for tumor phylogeny inference: tissue-wide and cell-by-cell. This new model is likely to benefit in the future from further methodological insights from both the unmixing and the phylogenetics fields.

## Methods

### Validation on simulated data

Our first simulated data protocol was designed to model uniformly sampled mixtures of components. In this protocol, we specify a dimension (number of genes) *d*, a number of samples *n*, a number of mixture components *k*, and a noise fraction σ. We then simulate *k *components by constructing a *d *× *k *matrix *C*^(*true*) ^in which each column is a mixture component and each element of that column is the expression of one hypothetical gene, sampled from a unit normal distribution. We then sample *n *mixture points by picking uniformly among possible fractional contributions of the components (*f*_*i*1_, ..., *f*_*ik*_) for each data point *i*, defining a mixture fraction matrix *F*^(*true*)^. Entries of the input data matrix *M *are then then derived from the formula

where *Z*_*ij *_is a unit normal random variable, implementing a log normal noise model. The resulting matrix *M *is then processed by the algorithm described in "Cell type identification by unmixing" above to infer some *C*^(*inferred*) ^and *F*^(*inferred*)^. To assess the quality of the assignment, we first match inferred mixture components to true mixture components by performing a maximum weighted bipartite matching of columns between *C*^(*true*) ^and *C*^(*inferred*)^, weighted by negative Euclidean distance. We then assess the quality of the mixture component identification by the root mean square distance over all entries of all components between the matched columns of the two *C *matrices:

We similarly assess the quality of the mixture fractions by the root mean square distance between *F*^(*true*) ^and *F*^(*inferred*)^over all genes and fractions:

This process was performed for *n *= 100 and *d *= 10, 000 to approximate a realistic tumor expression data set and evaluated for *k *= 3 to *k *= 7 and for σ = {0, 0.1, 0.2, ..., 1.0}, with ten repetitions per parameter.

Our second protocol was meant to model the assumption that each observed sample encodes a subset of an evolutionary tree. The protocol is parameterized by dimension *d*, number of samples *n*, number of mixture components *k*, and noise fraction σ, as with the uniform samples. The generation of the component matrix *C*^(*true*)^likewise proceeds exactly as with uniform samples. We assume, however, that each mixture component corresponds to one node in a binary evolutionary tree and that each observed sample corresponds to a path from the root to some arbitrary node in that tree. To generate the mixture fractions for a given simulated tumor sample, we select a tree node uniformly at random and then uniformly sample a set of mixture fractions of the chosen node and all of its ancestors in the tree, setting the mixture fraction to zero for all other components. After the generation of *C*^(*true*) ^and *F*^(*true*)^, the application of the inference methods and the evaluation of *C*^(*inferred*) ^and *F*^(*inferred*)^proceeds identically to that described for uniform mixture fractions in the preceding paragraph.

We additionally tested the accuracy of phylogeny inference on the tree-embedded samples. Following component inference and mixture fraction assignment, we applied the phylogeny inference algorithm described in "Phylogeny inference" above to infer trees on the components. For each test, we matched inferred to true components using maximum matching as above and used these assignments to determine the correspondence between edges present in the true trees and those present in the inferred trees. We scored the accuracy of each tree assignment as the fraction of true tree edges found in the inferred tree. For each condition, we recorded the average accuracy across repetitions.

### Application to real data

We retrieved the Jones et al. [33] lung cancer data from the Entrez GEO database [51] where it is indexed as dataset GSE1037. We then converted the data from log to linear scale by replacing each reported normalized expression value *x *with the value 2^*x*^. Missing values were aribtrarily assigned linear expression level 1. In order to minimize effects of outlier data points that are likely to be due to assay failures, we further restricted the lower and upper ranges of the data values to 2^-5^and 2^5^, respectively, setting values outside that range to the closer limit. This step was necessary because of the linearity assumption of the data, which would otherwise cause even a few extremely large values to dominate the calculations.

After processing the dataset, we then applied the unmixing methods as described in "Cell type identification by unmixing." We performed analysis for two different numbers of desired components, four and six. We chose four primarily to allow visual analysis of the solutions, as the resulting three-dimensional simplex is the largest we can directly visualize. We performed a second analysis with six components in order to explore how the methods perform with a more involved component set. While we did perform additional inferences for higher component numbers, we have no empirical basis for evaluating them once they begin to sub-divide the known tumor classes. In addition, the simulated results provide little ground for confidence in predictions about larger numbers of components. In the interests of space, we therefore do not report results beyond *k *= 6. For each of the two runs, we characterized the components by the average fractional assignment of components to each labeled tumor type. We also determined the vectors of gene expression levels relative to sample means corresponding to each component and report these inferred expression vectors sorted by relative expression for each component.

Finally, we performed phylogeny inference for both sets of inferences using the algorithm of "Phylogeny inference." For these data, we performed bootstrap replicates of the phylogeny inference stage of analysis to assess confidence in particular edges. We repeated the phylogeny inferences 10,000 times with each data point preserved 90% of the time per sample. We recorded the fraction of times each edge appeared across replicates to establish confidences on the predictions.

The human subjects work described in this section was considered by the Carnegie Mellon University Institutional Review Board and ruled exempt from human subjects requirements in accordance with 45 CFR 46.101(b)(4) due to its exclusive use of publicly available, anonymized patient data.

## Authors' contributions

RS and SS both participated in conceiving the approach used in this work, conceiving and designing validation experiments, and interpreting the results. RS carried out the algorithm development, coding, and execution of the experiments in this work. Both authors contributed to writing the manuscript.

## Supplementary Material

Additional file 1 **Marker genes for the lung cancer four-component inference**. This supplementary table provides relative expression levels inferred for each annotated microarray probe for the Jones *et al*. lung cancer data set for each of the four inferred mixture components. Columns of the table correspond to the component number, the gene ID, gene description, and relative expression level. Values are sorted by component and in decreasing order of relative expression level within each component. Click here for file

Additional file 2 **Marker genes for the lung cancer six-component inference**. This supplementary table provides relative expression levels inferred for each annotated microarray probe for the Jones *et al*. lung cancer data set for each of the six inferred mixture components. Columns of the table correspond to the component number, the gene ID, gene description, and relative expression level. Values are sorted by component and in decreasing order of relative expression level within each component. Click here for file
